# Association between Inflammation and Function of Cell Adhesion Molecules Influence on Gastrointestinal Cancer Development

**DOI:** 10.3390/cells10010067

**Published:** 2021-01-04

**Authors:** Hsiang-Wei Huang, Cheng-Chih Chang, Chia-Siu Wang, Kwang-Huei Lin

**Affiliations:** 1 Department of Biochemistry, College of Medicine, Chang Gung University, Taoyuan 333, Taiwan; memorywind@hotmail.com; 2 Department of General Surgery, Chang Gung Memorial Hospital at Chia-yi, Chia-yi 613, Taiwan; m7021@cgmh.org.tw (C.-C.C.); wangcs@cgmh.org.tw (C.-S.W.); 3 Liver Research Center, Chang Gung Memorial Hospital, Taoyuan 333, Taiwan; 4 Research Center for Chinese Herbal Medicine, College of Human Ecology, Chang Gung University of Science and Technology, Taoyuan 333, Taiwan

**Keywords:** gastrointestinal cancer, inflammation, tumor microenvironment, cell adhesion molecule

## Abstract

Gastrointestinal cancer is highly associated with inflammatory processes inducing the release of cytokines from cancer or immune cells, including interferons, interleukins, chemokines, colony-stimulating factors, and growth factors, which promote or suppress tumor progression. Inflammatory cytokines within the tumor microenvironment promote immune cell infiltration. Infiltrating immune, and tumor-surrounding stromal cells support tumor growth, angiogenesis, metastasis, and immunosuppression through communication with inflammatory cytokines and cell adhesion molecules. Notably, infiltrating immune and tumor cells present immunosuppressive molecules, such as programmed death-ligand 1 (PD-L1) and CD80/CD86. Suppression of cytotoxic T cells promotes tumor avoidance of immune surveillance and greater malignancy. Moreover, glycosylation and sialylation of proteins hyperexpressed on the cancer cell surface have been shown to enhance immune escape and metastasis. Cytokine treatments and immune checkpoint inhibitors are widely used in clinical practice. However, the tumor microenvironment is a rapidly changing milieu involving several factors. In this review, we have provided a summary of the interactions of inflammation and cell adhesion molecules between cancer and other cell types, to improve understanding of the tumor microenvironment.

## 1. Introduction

Metastasis is the major cause of cancer-related death. Several components of the tumor microenvironment contribute to metastatic progression, including angiogenesis, epithelial-mesenchymal transition (EMT), stromal cell support, immunocyte/inflammatory cell infiltration, cytokine/chemokine disorder, and cell–cell interactions. The majority of studies to date have focused on the intracellular molecular pathways of tumorigenesis. However, recent findings suggest that the extracellular environment of cancer cells is also important for tumor progression. For instance, tumor cells recruit immunocytes and inflammatory cells through secreted cytokines and chemokines [1]. Infiltrating immune cells secreteinterleukin-6 (IL-6), tumor necrosis factor-alpha (TNFα), transforming growth factor-beta (TGFβ), and growth factors to generate feedback loops that support tumor progression and metastasis [2], with subsequent infiltration of inflammatory cells, such as macrophages, which are differentiated into tumor-associated macrophage types (TAM) receiving different tumor-secreted factors (designated M1 and M2 macrophages) [3]. M1 macrophages kill invading pathogens and cancer cells by producing pro-inflammatory cytokines, such as IL-1β, IL-6, and TNFα [4]. Conversely, M2 macrophages promote tumor development by producing anti-inflammatory cytokines, such as IL-4, IL-10, IL-13, and TGFβ [5], providing an immunosuppressive microenvironment that promotes tumor immune escape [6]. Immunosuppression is mediated by cancer cells expressing programmed death-ligand 1 (PD-L1) and CD80 that bind PD-1 and cytotoxic T-lymphocyte-associated protein 4 (CTLA4) on cytotoxic T cells to suppress T cell activation [7]. Furthermore, tumor-secreted factors promote the generation of myeloid-derived suppressor cells (MDSC) for recruitment to tumor surroundings, which inhibit T cell activity [8]. Moreover, natural killer cells (NK cells) are suppressed by tumor-derived molecules, such as PD-L1 and prostaglandin E2 (PGE-2) [9]. Cytotoxic activity of NK cells can be inactive by TAMs and other immune cells. Tumor-infiltrating monocytes and macrophages suppress the function of NK cells by repress interferons-γ (IFNγ), TNFα, and Ki-67 expression of NK cells through TGFβ1 [10]. Cytokines and chemokines of the tumor microenvironment enhance both tumor immunosuppression and angiogenesis [11]. These results collectively suggest that the tumor microenvironment plays a critical role in tumor development through participation in immunosuppression and angiogenesis.

Chronic inflammation promotes tumorigenesis. Epidemiologic studies have shown that inflammation is a major risk factor for several gastrointestinal cancers, including colorectal, gastric, liver, pancreatic, and esophageal tumors types [12]. The relationship between colorectal cancer and inflammatory bowel disease (IBD) has been extensively documented. Inflammation is often associated with dysbiosis of the gastrointestinal microbiota, whereby changes in the bacterial population may cause an inflammatory reaction and contribute to tumorigenesis [13]. Inflammation caused by *Helicobacter pylori* infection is considered to be the main cause of gastric cancer since cytotoxin-associated gene A (CagA) seropositivity is significantly associated with disease risk [14]. Hepatic injury and inflammation are known risk factors for hepatocellular carcinoma (HCC). Chronic inflammation associated with carcinogenesis is predominantly due to viral infections, such as hepatitis B virus (HBV) and hepatitis C virus (HCV) [15].

Chronic inflammation induced field cancerization; the reason is mainly certain chemical effectors and molecular signals within the cell are continuously activated, such as oxidative stress, reactive oxygen species (ROS), cyclooxygenase-2 (COX-2), and inducible nitric oxide synthase (iNOS). These phenomena lead to DNA damage and somatic mutation that increases cancer risk. For instance, the imbalance between the accumulation of ROS and the production of antioxidants in cells leads to oxidative stress [16]. However, the component of ROS includes superoxide anion, hydrogen peroxide, and hydroxyl radical [17]. ROS has extremely high activity due to unpaired electron; therefore, it reacts strongly with any substance, including deoxyribonucleic acid (DNA).Thus, inflammation may also increase DNA damage and genomic instability by inducing ROS [18]. Besides, DNA damage has been increased the DNA mutation rate and trigger somatic mutation that increases the expression of an oncogene (such as the RAS family and Myc) or decreases the expression of tumor-suppressor genes (such as p53, PTEN, and Rb) [19]. On the other hand, COX-2 and iNOS are critical roles in inflammation and tumor initiation. COX-2 is also known as prostaglandin-endoperoxide synthase 2, which is the key enzyme in prostaglandin E2(PGE2) biosynthesis, whereas PGE2 overexpression is a sign of inflammation. This phenomenon suggests that COX-2 is involved in inflammation [20]. Moreover, COX-2 induces gastric cancer invasion and metastasis by reducing E-cadherin expression through nuclear factor-κB (NF-κB)/Snail signaling pathway [21]. In inflammatory cells, iNOS is highly activated, which regulates the conversion of L-arginine to L-citrulline and the release of nitric oxide (NO) free radicals [22]. NO has antimicrobial effects, but high concentrations of NO can cause cytotoxicity. In the past decades, NO was confirmed to be a key role in tumorigenesis and tumor progression [23]. Furthermore, iNOS has overexpression and correlation with tumor angiogenesis in gastrointestinal cancer [24,25]. Taken together, inflammation contributes to tumor initiation and regulates the tumor microenvironment, which increases malignancy in cancer.

The current review provides a detailed summary of the inflammation of gastrointestinal cancer development. Indicators of cell communication pathways in the tumor microenvironment that promote immunosuppression, angiogenesis, and metastasis that have been identified in documented studies are discussed.

## 2. Tissue Inflammation and the Tumor Microenvironment

Inflammation is a normal physiological response induced by harmful stimuli, including allergens, pathogens, toxic compounds, and injured tissues. Inflammatory responses present a mechanism of defense that can repair damage from foreign objects and promote wound healing [26]. Although the inflammatory response is induced by variable factors, common processes include stimulus recognition, inflammatory cytokine, and chemokine release, inflammatory cell recruitment, and activation of the inflammatory response [27]. Cancer cells effectively use components of inflammatory processes to create a suitable microenvironment for tumor growth, facilitating EMT, immune evasion, angiogenesis, and metastasis [28]. The major cytokines involved in inflammation include interferons, interleukins, chemokines, colony-stimulating factors, and growth factors.

### 2.1. Interferons

The physiological functions of interferons (IFNs) are to modify innate immunity and activate anti-viral/bacterial ability. Three types of IFNs have been identified, designated types Ι, ΙΙ, and ΙΙΙ, among which types I (α and β) and II (γ) have been shown to play important roles in cancer development [29]. IFNα and β bind to type I interferon receptor IFNAR1 and IFNAR2 that activate Janus kinase (JAK) and tyrosine kinase 2 (TYK2) signals, in turn, inducing transcription of interferon-stimulated genes (ISG) and signal transducer and activator of transcription (STAT) signaling pathways [30], which leads to enhanced T cell and NK cell-killing ability [31]. A recent study reported that type I interferons enhance the activity of tumor-infiltrating cytotoxic T lymphocytes (CTL) by activating the STAT3/granzyme B pathway. In colorectal carcinoma, IFNAR1 expression on CTLs is decreased to repress cell-killing activity [32]. In gastric cancer, IFNα inhibits c-casitas B-lineage lymphoma (c-Cbl) expression to activate the mitogen-activated protein kinase (MAPK)/extracellular signal-regulated kinase (ERK) pathway and subsequently induces tumor necrosis factor-related apoptosis-inducing ligand (TRAIL)-induced apoptosis [33]. Interferon-stimulated gene 15 (ISG15) conjugation promotes protein ISGylation and ubiquitination that upregulates IFNα-induced p53 and p21 expression to enhance HCC apoptosis [34]. Besides, IFNα restores the chemosensitivity (e.g., 5-fluorouracil, cisplatin, and gemcitabine) in pancreatic cancer [35] and IFNβ also enhances sensitivity to gemcitabine [36]. Type I interferons serve as critical mediators of the immune response and are used in combination anti-cancer treatments as potential adjuvants, including chemotherapy, targeted therapy, radiotherapy, and immunotherapy [37,38]. IFNγ binds interferon-γ receptor 1 (IFNGR1) and activates the JAK/STAT signaling pathway. Dual roles of IFNγ in tumor immunity have been reported, as either a suppressor or promoter of tumor development [39]. IFNγ increases integrin β3 expression via upregulating p50/p65 phosphorylation and induces gastric cancer cell proliferation and metastasis [40]. IFNγ may additionally enhance gastric cancer immunosuppression by inducing hepatocyte growth factor receptor (HGFR/MET)-mediated PD-L1 expression [41]. In pancreatic cancer, IFNγ increases the expression of PD-L1 and induces EMT through the STAT1 signaling pathway, which promotes immunosuppression and metastasis [42]. On the other hand, IFNγ treatment induced cell cycle arrest in the G1/S phases and suppressed cell proliferation in gastric cancer [43]. Moreover, IFNγ and nivolumab have synergistic effects that decrease PD-1 expression on CD8^+^ T cells. Thus, CD8^+^ T cells induced by IFN-γ and nivolumab that repress the growth of pancreatic cancer [44]. These findings support the critical roles of interferons in tumorigenesis.

### 2.2. Interleukins

Interleukins (ILs) play essential roles in immune and inflammatory responses and are involved in the maturation, differentiation, migration, and adhesion of immune cells. Interleukins were originally thought to be produced by immune cells, such as leukocytes and lymphocytes. Subsequent studies disclosed secretion by other cell types, including cancer cells [45]. Recent experiments suggest that ILs have important functions in tumor development and regenerate via positive feedback loops for enhanced impact. For instance, IL-1β, and IL-6 are highly expressed in colorectal cancer and infiltrating IL-1β receptor/IL-6 producing cells are increased in the stroma, which stimulates tumor cell growth [46]. IL-1α and IL-1β in the tumor microenvironment play critical roles in colorectal cancer growth by inducing tumor-elicited inflammation through IL-17A and IL-22 production in T cells [47]. Aberrant expression of sphingosine-1-phosphate receptor 1 (S1PR1) and STAT3 facilitates liver metastasis in colorectal cancer, which promotes IL-6 expression as well as MDSC expansion and accumulation via the IL-6/S1PR1/STAT3 axis signaling pathway [48]. IL-6/STAT3 signaling can promote pancreatic cancer growth and metastasis, which induce suppressor of cytokine signaling 3 (SOCS3) methylation through DNA methyltransferase 1 (DNMT1) [49]. Tumor stromal cells contribute to proliferation, angiogenesis, drug resistance, and metastasis, supporting tumor development. For instance, cancer-associated fibroblast (CAF)-derived IL-6 interacts with the IL-6 receptor to activate STAT3 and ERK1/2 signaling pathways that promote tumor growth in gastrointestinal cancer [50]. Besides, CAF-derived IL-8 enhances phosphoinositide 3 kinase (PI3K)/protein kinase B (Akt) signaling, inducing IKB and p65 phosphorylation that increases ABCB1 expression to promote cisplatin resistance in gastric cancer [51]. In HCC, microenvironmental thyroid hormone stimulates ISG20 expression that increases IL-8 expression and secretion, promoting angiogenesis via activation of JAK2/STAT3 phosphorylation in endothelial cells [52]. In addition to CAFs, tumor-associated macrophages (TAMs) exert tumor-promoting effects. For instance, TAM-derived IL-10 promotes proliferation and invasion in gastric cancer by activating the c-Met/STAT3 signaling pathway [53]. However, high levels of serum IL-6, IL-8, and IL-10 are positive correlations with poor prognosis in pancreatic cancer [54]. Besides the commonly characterized proinflammatory ILs, other ILs have carcinogenic functions. For example, IL-32 induces Akt phosphorylation that activates β-catenin to promote IL-8, vascular endothelial growth factor (VEGF), matrix metalloproteinase 2 (MMP2) and MMP9 expression, which contribute to gastric cancer progression, and metastasis [55]. Moreover, ILs are important signal transducers between cells and exist widely in plasma. IL-6, IL-8, and IL-10 have been identified as potential diagnostic biomarkers for gastrointestinal cancer, which are correlated with poor prognosis [56,57,58,59,60,61]. Thus, tumors are not only dependent on signal transduction, but also receive and regulate microenvironment-signaling molecules, such as interleukins, to promote progression.

### 2.3. Chemokines

Chemokines are a family of small chemoattractant cytokines that support the development and homeostasis of the immune system. The major functions of chemokines are chemotaxis and immunocyte differentiation [62]. Tumor cells can recruit and domesticate immune cell populations via tumor-derived chemokines. Populations of tumor-associated immune cells can promote tumor progression through paracrine signaling of some chemokines. For example, Wnt5a^+^ TAMs enhance C-C motif chemokine ligand 2 (CCL2) secretion through the calcium/calmodulin-dependent protein kinase II (CaMKII)-ERK pathway, which contributes to colorectal cancer growth, metastasis, and recruit infiltrating macrophages [63]. CCL2 is additionally reported to affect the chemotherapeutic response of gastric cancer cells. Recent studies have shown that gastric cancer-driven autocrine CCL2 activates PI3K/Akt/mTOR signaling to suppress proapoptotic autophagy, subsequently promoting sequestosome 1 (SQSTM1)-mediated CCL2 expression via NF-κB signaling. This positive feedback loop enhances chemotherapeutic resistance in gastric cancer [64]. Moreover, CCL2 induces angiogenesis through infiltrating macrophage-derived VEGF signaling in gastric cancer [65]. Long noncoding RNAs (lncRNAs) have attracted increasing research attention in recent years. The extensively characterized onco-lncRNA HOX transcript antisense intergenic RNA (HOTAIR) stimulates CCL2 expression along with the proliferation of macrophages and MDSCs in HCC, resulting in tumor progression [66]. These studies suggest that the CCL2 chemokine plays a critical role in the development of gastrointestinal cancer. Other chemokines known to regulate tumor development and progression include the CCR5 receptor ligands CCL3, CCL4, and CCL5. These chemokines, detected in sera of colorectal and gastric cancer patients, are correlated with poor prognosis [67,68]. CCR5 and CCL5 axis signaling induce F-actin polymerization in pancreatic cancer, which facilitates migration and invasion [69]. CCL3, CCL4, and CCL5 binding to the CCR5 receptor promote downstream signaling pathways that facilitate tumor cell proliferation, angiogenesis, metastasis, immune cell recruitment, and repolarization in gastrointestinal cancer [70,71]. The collective findings to date support the relevance of chemokines in the recruitment and domestication of immune cells to provide a suitable environment for tumor growth.

### 2.4. Colony-Stimulating Factors

Four types of colony-stimulating factors (CSFs) have been identified, specifically macrophage colony-stimulating factor (M-CSF), granulocyte-macrophage colony-stimulating factor (GM-CSF), granulocyte colony-stimulating factor (G-CSF), and multipotential colony stimulating factor (multi-CSF). CSFs are secreted glycoproteins that interact with colony-stimulating factor receptors (CSFRs) to activate downstream signaling pathways. CSFs play a key role in recruiting hematopoietic progenitor cells and stimulating granulocyte/macrophage differentiation [72]. Interestingly, GM-CSF induces macrophage differentiation into M1 macrophages that exert antitumor activity while M2 macrophage polarization by M-CSF contributes to tumor growth and metastasis [73]. Tumor cells not only secrete CSFs directly to regulate the balance of the microenvironment, but also stimulate infiltrating immune cells to secrete CSFs to further promote tumor development. Colorectal cancer-derived TNFα facilitates tumor growth and TAM recruitment, in turn, inducing TAM-mediated secretion of autocrine CSF1. Secreted CSF1 increases MMP2 and VEGF-A expression in TAMs, promoting tumor angiogenesis [74]. Moreover, components of the CSF1/CSF1R axis are highly expressed in gastric cancer and promote tumor proliferation and migration. The CSF1/CSF1R axis is positively correlated with VEGF-A expression, which contributes to tumor angiogenesis [75]. Among the four CSFs, GM-CSF that acts as a mediator of immune modulation and hematopoiesis is the most important contributor to tumor progression. GM-CSF is significantly involved in the differentiation, activation, and maturation of dendritic cells (DCs) and has been extensively employed as cancer immunotherapy in clinical trials (including monotherapy, GM-CSF-secreting cancer vaccines, GM-CSF-fused tumor-associated antigen (TAA) protein-based vaccines, and combination therapy). Clinical trials on GM-CSF are currently being conducted for several cancer types including melanoma, lymphoma, lung cancer, breast cancer, gastrointestinal cancer, and ongoing phase I-III trials support its utility as a potential vaccine for cancer immunotherapy [76,77,78]. Several previous studies indicate that GM-CSF acts as a tumor-promoting factor that contributes to proliferation, angiogenesis, immunosuppression, and metastasis. For instance, colorectal cancer-derived GM-CSF induces VEGF secretion by colonic epithelial cells through an ERK signaling pathway, which promotes tumor proliferation, angiogenesis, migration, and invasion [79]. Moreover, chronic stimulation by GM-CSF induces EMT in colorectal cancer, promoting metastasis, and chemoresistance [80]. In HCC, the activated NF-κB pathway enhances GM-CSF and IL-6 secretion by tumor and tumor-associated endothelial cells, respectively, which facilitate MDSC recruitment. Infiltrating MDSCs promote tumor growth and metastasis via inhibition of Th1 CD4^+^ and cytotoxic CD8^+^ T cell activities and increased angiogenesis, but this phenotype can be blocked by chemerin [81]. GM-CSF provides a suitable microenvironment for immune evasion in gastrointestinal cancer. PD-L1 is a well-established target for immunosuppression. Recent studies have shown that HCC and gastric cancer-derived GM-CSF bind neutrophil receptors and stimulate JAK2 and STAT3 phosphorylation that promotes expression of PD-L1 on the neutrophil surface, which binds and inhibits PD-1 on cytotoxic T cells [82,83,84]. In pancreatic ductal adenocarcinoma (PDAC), HIF-1α direct regulates GM-CSF promoter activity that induces the expression and secretion of GM-CSF. The HIF-1α/GM-CSF promotes the occurrence of perineural invasion, leading to poor prognosis of PDAC patients [85]. Thus, CSFs play critical roles in tumor progression and development.

### 2.5. Growth Factors

In addition to IFNs, ILs, CSFs, and chemokines, growth factors (GFs) are involved in inflammation, including TNFα, TGFβ, and platelet-derived growth factor (PDGF). TNFα is a proinflammatory cytokine mainly secreted by macrophages that functions in the mediation of the immune response contributing to inflammation, anti-viral, apoptosis, and antitumor activities. Disruption of TNFα production underlies numerous diseases, including inflammatory bowel disease (IBD) and cancer. In HCC, infiltrating M2-TAM-derived TNFα promotes EMT and cancer stemness via the Wnt/β-catenin signaling pathway [86]. EMT induced by TNFα contributes to sorafenib resistance in HCC [87]. NADPH oxidase 1 (NOX1)-induced reactive oxygen species (ROS) signaling contributes to gastric cancer development and stemness through effects on the TNFα/NF-κB pathway [88]. Notably, gastric cancer-derived TNFα induces PD-L1 expression of intratumoral mast cells via NF-κB signaling that inhibits T cell activity [89], further supporting its role as a tumor-promoting factor. Another important proinflammatory factor is TGFβ. Activation of TGFβ receptors by TGFβ binding turns on downstream signaling through classical (SMAD) or non-classical (PI3K/MAPK) pathways. Three SMAD types exist, specifically, receptor-regulated SMADs (R-Smads), common partner SMADs (Co-Smads), and inhibitory SMADs (I-Smads). R-Smads include Smad1, Smad2, Smad3, Smad5, Smad8, and Smad9 that are activated by the TGFβ receptor. Smad4 is a co-Smad that co-regulates TGFβ signaling with R-Smads. Smad6 and Smad7 are I-Smads, which inhibit R-Smad-mediated signaling [90]. Numerous studies have documented TGFβ-mediated enhancement of tumor growth, angiogenesis, metastasis, and immunosuppression. For example, TGFβ induces angiogenesis and EMT of colorectal cancer through c-Jun N-terminal kinase (JNK) signaling and is negatively regulated by p38α of mesenchymal cells [91]. Neutrophil-secreted MMP9 activates TGFβ signaling, leading to suppression of T cell activity in colorectal cancer [92]. In addition to angiogenesis, TGFβ enhances gastric cancer-induced lymphangiogenesis through Smad2/3-mediated VEGF-C signaling [93], mediates Smad3 phosphorylation, and promotes protein tyrosine phosphatase receptor epsilon (PTPRε) transcription, leading to migration, invasion, and metastasis of HCC [94]. Chemoresistance in HCC is induced by TGFβ through the ERK pathway, which promotes the pregnane X receptor (PXR) expression [95]. Interestingly, TGFβ-mediated EMT induces N-glycosylation of CD147/basigin by N-acetylglucosaminyltransferase V (GnT-V) that enhances interactions with integrinβ1 and promotes HCC metastasis [96]. Furthermore, TGFβ1 and GM-CSF suppress chemotherapeutic effects by modulating the tumor microenvironment in pancreatic cancer [97]. These findings suggest that both secreted protein and protein-protein interactions on the cell surface are critical for tumor progression. Chronic inflammation triggers cytokine disorders often accompanied by angiogenesis. VEGF and PDGF are known as critical angiogenic factors. The PDGF glycoprotein can exist as a homodimer or heterodimer (PDGF-AA, PDGF-BB, or PDGF-AB). Biological functions of PDGFs include regulation of cell proliferation, progenitor cell differentiation, and blood vessel formation. Angiogenesis is a necessary process for tumorigenesis that also involves PDGFs. Recent data suggest that thyroid hormone stimulates nuclear protein-1 (NUPR1) expression, with subsequent NUPR1-mediated activation of PDGFA transcription via direct targeting of the promoter in HCC. HCC-derived PDGFA induces angiogenesis through MEK/ERK signaling, which can be blocked by sorafenib [98]. Mutant p53 abnormal interact with p73 that NF-Y complex sustained activate PDGF receptorβ (PDGFRb), leading to metastasis in pancreatic cancer [99]. Based on the collective results, it is proposed that cancer cells are further activated by inflammatory factors and chronic inflammation thus provides an ideal environment for tumor development (Table 1).

## 3. Molecular Communication between Tumor and Other Cells in the Tumor Microenvironment

In addition to inflammatory factors, cell-cell interactions are important for tumor progression. Tumor cells express specific molecules on their surfaces that facilitate interactions with immune, endothelial, and other types of cells. On the other hand, tumor-derived inflammatory factors activate tumor-associated cells presenting specific molecules that promote tumor development, such as cadherins, selectins, integrins, CD80, and PD-L1, which undergo modifications, such as glycosylation or sialylation, to enhance or repress the interactions. These interactions contribute to tumor immunosuppression and metastasis.

### 3.1. Cadherins

Cadherins are dependent on calcium ions to perform their functions, so they are also named calcium-dependent adhesion. Cadherins play a critical role in the formation of zonula adherens that regulating cell-cell adhesion [100]. During development, cadherins contribute to properly positioning cells and assist the separation of the different germ layers [101]. However, many studies indicate that cadherins is an important role in tumor metastasis via EMT, which promotes tumor progression. For example, epigenetic repress E-cadherin expression by enhancer of zeste homolog 2 (EZH2) and histone deacetylases 6 (HDAC6), which are increased through Slug signaling that facilitating EMT and metastasis in colorectal cancer [102]. Moreover, E-cadherin dysfunction in liver cancer and gastric cancer correlates with tumorigenesis and metastasis [103,104]. These results suggest that E-cadherin is a tumor tumor-suppressor gene, which is well known as an epithelial marker. In turn, N-cadherin is an oncogene, which is well known as a mesenchymal marker. For instance, STAT3 regulated zinc finger E-box-binding homeobox 1 (ZEB1) expression that decreased E-cadherin and increased N-cadherin/vimentin expressions, which promote metastasis in colorectal cancer [105]. In gastric cancer, metastasis is facilitated by TGF-β1/Snail signaling that decreased expression of E-cadherin and increased expression of N-cadherin [106]. On the other hand, vascular endothelial cadherin (VE-cadherin) is major involved in vascular permeability, endothelial cell proliferation, and capillary formation, which relate to angiogenesis. LncRNA MALAT1 induces metastasis of gastric cancer by facilitating angiogenesis through the VE-cadherin/β-catenin, ERK/MMP, and FAK/paxillin signaling pathways [107]. Thus, cadherin is an important role in tumor metastasis.

### 3.2. Selectins

Selectins are transmembrane glycoproteins that dependent on calcium ions. They are the family of cell adhesion molecules (CAM), including endothelial selectin (E-selectin), leukocyte selectin (L-selectin), and platelet selectin (P-selectin). Selectins mediate physiological responses, such as leukocyte rolling, adhesion, migration, inflammation, and hemostasis [108]. Indeed, selectins also mediate pathological inflammation, immune surveillance, and tumor progression. Many studies indicate that selectins have an important role in tumor cell adhesion, and intravasation into the blood vessel, and regulate immune responses within a tumor microenvironment [109]. For example, IL-1β induces E-selectin-dependent extravasation of colon cancer cells, and subsequently regulating E-selectin expression by modulating miR-31 transcription [110]. Inhibition of E-selectin expression of endothelial cells may reduce endothelial and tumor cell adhesion, which inhibits HCC growth and angiogenesis [111]. Moreover, P-selectin binding to colon cancer cells, which activate integrin α5β1 through PI3K and p38 MAPK signaling pathways that increased cell adhesion and metastasis [112]. In the mouse colon adenocarcinoma model, P-selectin facilitates metastasis by regulating platelets and tumor cell interaction. Surprisingly, L-selectin of leukocytes also facilitates metastasis. It means P-selectin and L-selectin have synergistic effects in the tumor metastatic process [113]. The results obtained to date suggest that selectin contributes to the tumor cell and other types of cell interaction, which facilitates tumor metastasis.

### 3.3. Integrins

Integrins, which exist as heterodimers composed of α and β subunits, are transmembrane receptors. Cell adhesion to the extracellular matrix (ECM) is promoted through integrin regulation [114]. Integrin binding with ECM mediates cytoskeleton remodeling and activates intracellular signaling, in turn, regulating cell growth, spreading, migration, and differentiation [115]. Integrins overexpressed in gastrointestinal cancer are known to regulate tumor progression. For instance, ECM-mediated integrin β1 promotes liver metastasis of gastrointestinal cancer through focal adhesion kinase (FAK)-yes-associated protein 1 (YAP1)/tafazzin (TAZ) signaling, which is enhanced by vasodilator-stimulated phosphoprotein (VASP) [116]. In addition, ECM1-mediated integrin β4 promotes EMT and glucose metabolism via SOX2/HIF-1α signaling in gastric cancer [117] and modulates epidermal growth factor receptor (EGFR) signaling to induce gefitinib chemoresistance [118]. In pancreatic cancer, solute carrier family 39 member 4 (ZIP4) induces ZEB1 expression via STAT3 signaling and subsequently activate integrin α3β1 to regulate the JNK signaling pathway, leading to inhibit chemosensitivity by decrease gemcitabine transporter ENT1 [119]. Recent studies have shown that integrins are activated by cellular adhesion molecules, cytokines, and other stimuli. In colorectal cancer, cadherin-17 (CDH17) interacts with integrin α2β1 to activate FAK and Ras pathways promoting proliferation and metastasis [120]. IFN-γ increases proliferation and metastasis via integrin β3-mediated NF-κB signaling in gastric cancer [40]. Chemokine (C-X-C motif) ligand 1 (CXCL1) secreted by tumor-associated lymphatic endothelial cells induces integrin β1 to activate FAK/Akt signaling and promotes adhesion and metastasis of gastric cancer via MMP2/9 upregulation [121]. In HCC, IL-8 promotes invasive ability via the integrin β3-mediated PI3K/Akt pathway [122]. Gastrointestinal cancer is an inflammation-related disease. Lipopolysaccharide (LPS), one of the main cell membrane components of gram-negative bacteria, is a well-known endotoxin that stimulates inflammation. LPS-induced toll-like receptor 4 (TLR4) signaling activates the integrin β1/PI3K/Akt axis, which enhances colorectal cancer cell adhesion and liver metastasis [123]. The majority of studies in recent years have focused on the involvement of glycosylation and sugar-binding proteins, such as galectin, in cancer progression. In HCC, galectin-1 (Gal-1) induces EMT and sorafenib resistance by increasing integrin αvβ3 expression that activates FAK/PI3K/Akt signaling [124]. PDAC-derived galectin-3 (Gal-3) induces production of IL8 through integrin/NF-κB signaling from stromal cells, which mediate remodeling of TME and promote tumor growth and metastasis [125]. Taken together, the data support the critical roles of cancer cell adhesion molecules in tumor growth, metastasis, and chemoresistance (Table 2).

### 3.4. Programmed Death-Ligand 1

Programmed death ligand 1 (PD-L1) is a B7 family member, also designated cluster of differentiation 274 (CD274) or B7 homolog 1 (B7-H1), whose receptor is programmed cell death 1 (PD-1). PD-L1 binds its receptor PD-1 to suppress the activity of the immune system and modulates the immune response under normal physiological conditions. PD-1 is predominantly expressed in activated CD4^+^ T cells, CD8^+^ T cells, natural killer T cells (NKT cells), B cells, NK cells, and activated monocytes, while PD-L1 in MDSCs, neutrophils, B cells, and DCs. PD-L1 expressed on the surface of tumor cells and TAMs promotes suppression of cytotoxic T cell activity and performs a protective function [126]. Clinically, high PD-L1 expression is associated with poor clinical outcomes in gastrointestinal cancer [127,128,129]. PD-L1 expression is regulated by either intracellular molecular mechanisms or extracellular stimulation. For example, the inflammatory cytokines TNFα and IL-17 enhance PD-L1 expression in colorectal cancer through activation of Akt, NF-κB, and ERK1/2 signaling [130]. On the other hand, LPS stimulates NF-κB binding to the PD-L1 promoter and enhances its transcription in gastric cancer [131]. Immune cells recruited by the tumor additionally support immune surveillance. Tumor-infiltrating lymphocyte (TIL)-driven IFNγ stimulates the JAK2/STAT1 pathway, leading to increased PD-L1 expression in gastric cancer [132]. Importantly, gastric cancer-driven GM-CSF induces high PD-L1 expression in tumor-infiltrating neutrophils and TAMs via JAK/STAT3 and IL-8 signaling, respectively. Moreover, GM-CSF facilitates TAM secretion of IL-8, which inhibits CD8+ T cell proliferation and infiltration [84,133]. Another interesting finding from a recent study is that PD-L1 is not only expressed in cells but also on the exosome surface. Circulating exosomal PD-L1 has a strong potential to induce immunosuppression in gastric cancer [134]. PD-L1 is therefore a critical biological interface for the immune escape of tumors.

### 3.5. Cytotoxic T-Lymphocyte Associated Protein 4

Cytotoxic T-lymphocyte-associated protein 4 (CTLA4) and CD28 co-regulate T cell activation. CTLA4 is a receptor constitutively expressed in regulatory T cells (Tregs) whereas CD28 is expressed in T cells. Their ligands, CD80 (B7-1), and CD86 (B7-2) are B7 family members expressed in antigen-presenting cells (APC). CTLA4 and CD28 induce inhibition and activation of T cells, respectively. CTLA4 has a higher binding affinity than CD28 for CD80 and CD86. Overexpression of CTLA4 on the Treg surface and competitive binding to CD80 and CD86 can impair T cell activity [135]. Antibody-based blockade of CTLA4 binding to CD80/CD86 is reported to promote CD80/CD86 interactions with the immune co-stimulatory signal, CD28, and re-activation of T cell activity to kill cancer cells [136]. CTLA4 and PD-1 are two important immune checkpoint molecules in cancer immunology. Importantly, tumors can escape immune surveillance through CTLA4 activity. For instance, ligands of CTLA4 and PD-1 highly expressed in colorectal cancer suppress T cell activity and maintain tumor Tregs. However, adoptive transfer of CD8^+^ TILs with dual antibody blockade of PD-1 and CTLA-4 in vitro still demonstrates killing tumor ability in vivo [137]. Cross-database analysis indicates that CTLA4 activation leads to immunosuppression and promotes the development of gastric cancer, contributing to poor prognosis [138].

### 3.6. Glycosylation

Post-translational modification (PTM) of proteins is an important regulatory mechanism for cell signal transduction and cell-cell cross talk. Several enzymatic steps are involved in glycosylation, considered the most complex PTM. In addition to materials, such as carbohydrates, glycosylation requires glycosyltransferases (GTs) and glycosidases. GTs are involved in glycan chain synthesis and glycosidases in specific glycan linkage hydrolysis. Five types of glycosylation have been identified that mainly occur in Golgi, specifically, N-linked glycosylation, O-linked glycosylation, phosphoserine glycosylation, C-mannosylation, and glypiation. Among these, N-linked and O-linked glycosylation are the most prevalent types. The carbohydrate binds the amine group of asparagine (designated N-linked) or hydroxyl groups of serine or threonine (designated O-linked). Cell surface glycoproteins are involved in cell adhesion, cell recognition and, ligand/receptor activation, among other processes [139]. In recent years, glycosylation has received significant attention in relation to cancer development. Indeed, glycoproteins play critical regulatory roles in tumor progression, including proliferation, angiogenesis, invasion, metastasis, inflammation, and immune escape [140,141]. For example, in colorectal cancer, VEGFR2 shows a higher modification of β1-6GlcNAc-branched N-glycans on endothelial cells that bind Gal-1, which, like VEGF, induces signaling. On the other hand, modification of α2-6-linked sialic acid prevents Gal-1 signaling, leading to anti-VEGFR2 activation [142]. O-GlcNAc transferase (OGT) catalyzes the process of O-GlcNAcylation. Silencing of OGT reduces O-GlcNAcylation levels and increases N-glycosylation of E-cadherin, which leads to enhanced cell junctions and suppression of migration in colorectal cancer [143]. Core 1 β1,3-galactosyltransferase (C1GALT1), overexpressed in HCC, promotes invasive ability through modifying O-glycosylation of integrin β1 [144]. Glycosylation is therefore an important factor for consideration in cancer development, which can enhance or suppress cell communication. Even if levels of molecules remain unchanged, glycosylation may cause alterations in signal transduction.

### 3.7. Sialylation

The majority of sialic acids are present at the end of oligosaccharide chains on glycoproteins or linked with galactose and N-acetylglucosamine on glycolipids. Sialic acid is linked to glycoproteins and glycolipids by different glycosidic bonds (α2-3, α2-6, or α2-8). Sialylation is an important glycosylation modification at the end of the oligosaccharide chain, with N-acetylneuraminic acid (Neu5Ac) and N-glycolylneuraminic acid (Neu5Gc) being the major substrates of sialyltransferases (STs). Neu5Ac is hydroxylated to generate Neu5Gc by cytidine monophospho-N-acetylneuraminic acid hydroxylase (CMAH). Loss of CMAH function in humans is mediated by an Alu-mediated frameshift deletion [145]. Thus, native Neu5Gc does not exist and is considered an oncofetal antigen that induces chronic inflammation and tumorigenesis [146]. Sialyltransferases are a family of 20 members including ST3Gal I-VI (α2-3-linkage), ST6Gal I-II (α2-6- and N-linkage), ST6GalNAc I-VI (α2-6- and O-linkage), and ST8Sia I-VI (α2-8-linkage) [147]. The biological functions of sialic acids include cell recognition, adhesion, and signal transduction. Sialic acids can modify several hormones, lectins, ligands, and receptors to mediate binding affinity. As a cell recognition molecule, sialic acid is used for either recognition or masking of a binding site. Cell surface sialylation regulates adhesion processes, such as immune cell adhesion in the immune response or viral infection. Importantly, sialic acids are a family of nine negatively charged carbon acidic monosaccharides that impart a negative charge to modified molecules. Repulsion or attraction caused by the negative charge of sialic acid plays an influential role in gastrointestinal cancer development [148,149]. Abnormal cell surface sialylation is also involved in cancer development, including migration, invasion, metastasis, and immune escape. For example, ST6Gal1-mediated α2,6-sialylation promotes proliferation and migration of HCC through CD147/MMP signaling while inducing immune escape by inhibiting T cell proliferation via increased secretion of IL-10 and TGFβ1 [150]. Silencing of ST6GalNAc6 is induced by the NF-κB-mediated YY1/PRC2/EZH2 axis, which binds to its promoter and represses activity, leading to loss of immunosuppression and promotion of colorectal cancer progression [151]. ST6Gal1 increases α2,6-sialylation of human epidermal growth factor receptor 2 (HER2) and activates PI3K/Akt and ERK pathways, which promote proliferation, adhesion, invasion, and suppress apoptosis of tumor cells in gastric cancer [152]. The results obtained to date suggest that sialic acids and sialyation can alter phenotypes of tumors via modification of transducing molecular and cell surface proteins. However, in view of the negatively charged characteristics of sialic acids, further research considering the impacts of positive and negative charges is required.

## 4. Immunotherapy

In addition to traditional surgery and chemotherapy, immunotherapy has been increasingly used in clinical treatments. The prognosis of patients can be effectively improved by combining with other therapies. Distinct from traditional therapies, cancer immunotherapy not only targets the tumor but also activates the immune response and creates an anti-tumor environment. Therapeutic approaches employing cytokines, immune checkpoint inhibitors, and chimeric antigen receptor T cells (CAR-T) are commonly employed. Cytokines, such as IFNα, IFNγ, IL-2, and GM-CSF, which induce B cells, T cells, NK cells, and macrophages for tumor cell killing, have been successfully used for gastrointestinal cancer treatment [153]. The two immune checkpoint inhibitors target PD-1/PD-L1 and CTLA4. Blockage of the PD-1/PD-L1 or CTLA4 signal with antibodies prevents binding of immunosuppressive molecules to cytotoxic CD8^+^ T cells, which maintains cytotoxic CD8^+^ T cell activity against cancer cells [154]. CAR-T therapy involves the collection of T cells from patients, adding chimeric antigen receptors that recognize tumor cells, and finally returning fused CAR-T cells to the patients to attack tumors [155,156]. However, CAR-T therapy is associated with a number of side-effects, among which cytokine storm is the most significant [157]. Inflammation triggers the release of multiple cytokines. When individuals with strong immune responses are infected with viruses or other pathogens, immune cells release inflammatory cytokines on a large scale, promoting macrophage and neutrophil activities to strengthen defense capability against foreign objects. However, excessive cytokine generation can lead to abnormal signal transduction and immune response disorders, ultimately resulting in an indiscriminate attack of immune cells, multiple organ failure, and death. This vicious cycle is known as “cytokine storm” [158]. Clinically, acute symptoms of viral infections are not caused by massive virus replication but overreaction of the immune system and release of large amounts of cytokines. Significant examples of this phenomenon are severe acute respiratory syndrome (SARS) and coronavirus disease 2019 (COVID-19) [159]. During CAR-T therapy, in rare cases, macrophages release a large number of cytokines, including IL-1 and IL-6, which can trigger a cytokine storm. Thus, inflammatory cytokines serve as a double-edged sword, acting as tumor promoting or tumor suppression factors, depending on the cell source or PTM. Now, safe CAR-NK therapy has been shown in clinical trials. Cytokine storm is not common in CAR-NK therapy compared to CAR-T therapy [160]. Taken together, inflammatory cytokines and cell adhesion molecules are critical roles in tumor development and cancer immunotherapy.

## 5. Conclusions

**Figure 1:**
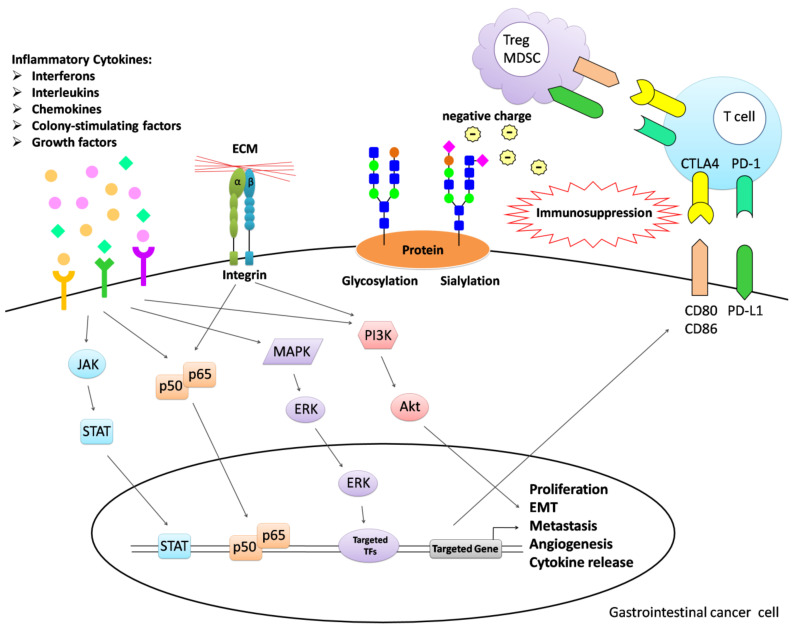
Overview of cell–cell communication in the tumor microenvironment. Inflammatory cytokines facilitate tumor proliferation, angiogenesis, metastasis, and immunosuppression through NF-κB, JAK/STAT, PI3K/Akt, and MAPK/ERK pathways. Integrins acting as cell adhesion molecules additionally contribute to the tumor phenotype. Infiltrating immune and tumor cells present CD80/CD86 and PD-L1, leading to loss of T cell activity. Glycosylation and sialylation on the cell surface promote metastasis and immune escape. Notably, the negative charges imparted by sialic acids enhance this phenomenon.

Inflammation-induced cytokine release is important for the tumor microenvironment and its complex composition is regulated by several pro- and anti-inflammatory factors in a dynamic balance. Different inflammatory cytokines with inherent characteristics of pleiotropism and redundancy may regulate the same signaling pathway. NF-κB, JAK/STAT, PI3K/Akt, and MAPK/ERK are the common signaling pathways for inflammatory cytokine stimulation. In the tumor microenvironment, tumor progression is affected by not only single cytokines, but also combinations of cytokines that exert additive, synergistic, or antagonistic effects to regulate cell proliferation, angiogenesis, metastasis, and immune surveillance. Cell adhesion molecules are additionally involved in these phenotypes (Figure 1). As mentioned earlier, understanding the interactions between inflammatory cytokines and cell adhesion molecules for promoting localization of the tumor and infiltrating cells in the tumor microenvironment is critical. These molecules usually undergo glycosylation and sialylation, suggesting that more complex factors require consideration. Recently, N-linked glycan modification of immunoglobulin gamma (IgG) at asparagine297 in the crystallizable fragment (Fc) region was reported to trigger autoimmune disease. Sialylation of N297 has been achieved by bioengineering, which imparts anti-inflammatory properties to self-reactive IgGs [161]. The hypothesis that inflammatory cytokines play dual roles in tumor development and maybe affected by glycosylation and sialylation requires further exploration. Glycosylation and sialylation on the surface of cancer cells clearly aid in the avoidance of immune cells and removal of sialylation has been shown to suppress tumor progression in breast cancer and melanoma [162,163]. Detailed elucidation of the biological interface of gastrointestinal cancer should facilitate the determination of tumor properties and the development of optimized clinical therapeutic strategies.

## Figures and Tables

**Table 1:** Inflammatory cytokines and their potential mechanisms in gastrointestinal cancer.

Cancer Types	Gene Name	Molecules and Signaling Pathways Involved	Principal Function	Ref.
**Interferons**				
**Tumor Suppressing Effects**				
Colorectal cancer	IFNα/β	STAT3/granzyme B pathway	Enhance the activity of CTL	[32]
Gastric cancer	IFNα	MAPK/ERK pathway	Apoptosis	[33]
	IFNγ		Inhibit proliferation	[43]
Liver cancer	IFNα	p53 and p21	Apoptosis	[34]
Pancreatic cancer	IFNα		Enhance chemosensitivity	[35]
	IFNγ	PD-1	Repress tumor growth	[44]
**Tumor Promoting Effects**				
Gastric cancer	IFNγ	integrin β3 p50/p65 pathway	Proliferation, Metastasis	[40]
		HGFR/MET/PD-L1 pathway	Immunosuppression	[41]
Pancreatic cancer	IFNγ	PD-L1 STAT1 signaling pathway	Immunosuppression, Metastasis	[42]
**Interleukins**				
Colorectal cancer	IL-1β and IL-6		Promote tumor growth	[46]
	IL-1α and IL-1β	IL-17A and IL-22 production	Promote tumor growth	[47]
	IL-6	IL-6/S1PR1/STAT3 axis signaling pathway	Metastasis	[48]
Pancreatic cancer	IL-6	STAT3/DNMT1/SOCS3	Proliferation, Metastasis	[49]
Gastric cancer	IL-8	PI3K/Akt pathway, IKB/p65 pathway, ABCB1	Cisplatin resistance	[51]
	IL-10	c-Met/STAT3 signaling pathway	Proliferation, Invasion	[53]
	IL-32	Akt, β-catenin, VEGF, MMP2, MMP9	Metastasis	[55]
Liver cancer	IL-8	ISG20, JAK2/STAT3 pathway	Angiogenesis	[52]
**Chemokines**				
Colorectal cancer	CCL2	CaMKII-ERK pathway	Promote tumor growth, Metastasis	[63]
	CCL3, CCL4, and CCL5.		Poor prognosis	[67]
Gastric cancer	CCL2	PI3K/Akt/mTOR signaling, SQSTM1, VEGF	Chemotherapeutic resistance, Angiogenesis	[64,65]
	CCL3, CCL4, and CCL5.		Poor prognosis	[68]
Liver cancer	CCL2	HOTAIR	Proliferation of macrophages and MDSCs	[66]
Pancreatic cancer	CCL5	F-actin	Migration, Invasion	[69]
**Colony-stimulating factors**				
Colorectal cancer	M-CSF	MMP2, VEGF-A	Angiogenesis	[74]
	GM-CSF	VEGF, ERK	Proliferation, Angiogenesis, Migration, Invasion, chemoresistance	[79,80]
Gastric cancer	M-CSF	VEGF-A	Promote tumor growth, Migration, Angiogenesis	[75]
	GM-CSF	JAK/STAT3 signaling pathway, PD-L1	Immunosuppression	[83,84]
Liver cancer	GM-CSF	NF-κB pathway, JAK/STAT3 signaling pathway, PD-L1	Promote tumor growth, Metastasis, Angiogenesis, Immunosuppression	[81,82]
Pancreatic cancer	GM-CSF	HIF-1α	Invasion, Chemoresistance	[85,97]
**Growth factors**				
Liver cancer	TNFα	Wnt/β-catenin signaling pathway	EMT, Cancer stemness, Sorafenib resistance	[86,87]
	TGFβ	PTPRε, ERK/PXR pathway, GnT-V/integrinβ1	Metastasis, Chemoresistance	[94,95,96]
	PDGFA	NUPR1, MEK/ERK signaling	Angiogenesis, Sorafenib resistance	[98]
Gastric cancer	TNFα	NF-κB pathway, NOX1, PD-L1	Cancer stemness, Immunosuppression	[88,89]
	TGFβ	Smad2/3 signaling pathway, VEGF-C	lymphangiogenesis	[93]
Colorectal cancer	TGFβ	JNK, p38, MMP9	Angiogenesis, Immunosuppression	[91,92]
Pancreatic cancer	TGFβ1		Chemoresistance	[97]
	PDGFB	p53, PDGFRb	Metastasis	[99]

**Table 2:** Cell adhesion molecules and their potential mechanisms in gastrointestinal cancer.

Cancer Types	Gene Name	Molecules and Signaling Pathways Involved	Principal Function	Ref.
**Cadherins**				
Colorectal cancer	E-cadherin	EZH2, HDAC6, Slug	EMT, Metastasis	[102]
	N-cadherin	STAT3, ZEB1, Vimentin	Metastasis	[105]
Liver cancer	E-cadherin		Tumorigenesis	[103]
Gastric cancer	E-cadherin		Metastasis	[104]
	N-cadherin	TGF-β1/Snail signaling	Metastasis	[106]
	VE-cadherin	LncRNA MALAT1, β-catenin, ERK/MMP and FAK/paxillin signaling pathways	Angiogenesis	[107]
**Selectins**				
colon cancer	E-selectin	IL-1β, miR-31	Extravasation	[110]
	P-selectin	integrin α5β1, PI3K and p38 MAPK signaling pathway	Cell adhesion, Metastasis	[112]
	L-selectin		Metastasis	[113]
Liver cancer	E-selectin		Tumor growth, Angiogenesis	[111]
**Integrins**				
Gastric cancer	integrin β4	SOX2/HIF-1α signaling, EGFR	EMT, Glucose metabolism, gefitinib chemoresistance	[117,118]
	integrin β1	CXCL1, FAK/Akt signaling, MMP2/9	Adhesion, Metastasis	[121]
Pancreatic cancer	integrin α3β1	ZIP4, ZEB1, STAT3 signaling, JNK signaling pathway	Chemoresistance	[119]
	Integrin family	galectin-3, IL-8, NF-κB signaling	Promote tumor growth, Metastasis	[125]
Colorectal cancer	integrin α2β1	CDH17, FAK and Ras pathways	Proliferation, Metastasis	[120]
	integrin β1	TLR4, PI3K/Akt pathway	Adhesion, Metastasis	[123]
Liver cancer	integrin β3	IL-8, PI3K/Akt pathway	Invasion	[122]
	integrin αvβ3	galectin-1, FAK/PI3K/Akt signaling	EMT, Sorafenib resistance	[124]

## Data Availability

Not applicable.
